# Process Evaluation of a Multidisciplinary Care Program for Patients Undergoing Gynaecological Surgery

**DOI:** 10.1007/s10926-013-9475-4

**Published:** 2013-09-22

**Authors:** E. V. A. Bouwsma, A. Vonk Noordegraaf, Z. Szlávik, H. A. M. Brölmann, M. H. Emanuel, J. P. Lips, W. van Mechelen, A. Mozes, A. L. Thurkow, J. A. F. Huirne, J. R. Anema

**Affiliations:** 1 Department of Public and Occupational Health, EMGO Institute for Health and Care Research, VU University Medical Center, Amsterdam, The Netherlands; 2 Department of Obstetrics and Gynaecology, VU University Medical Center, Amsterdam, The Netherlands; 3 Department of Computer Science, VU University, Amsterdam, The Netherlands; 4 Department of Obstetrics and Gynaecology, Spaarne Hospital, Hoofddorp, The Netherlands; 5 Department of Obstetrics and Gynaecology, Kennemer Gasthuis, Haarlem, The Netherlands; 6 Department of Obstetrics and Gynaecology, Amstelland Hospital, Amstelveen, The Netherlands; 7 Department of Obstetrics and Gynaecology, Sint Lucas Andreas Hospital, Amsterdam, The Netherlands

**Keywords:** Gynaecology, Telemedicine, Convalescence, Return to work, Program evaluation

## Abstract

*Purpose* This study describes the process evaluation of an innovative multidisciplinary care program for patients undergoing benign gynaecologic surgery. This care program aims at improving recovery and preventing delayed return to work and consists of two steps: (1) an interactive e-health intervention for all participants, and (2) integrated clinical and occupational care management for those participants whose sick leave exceeds 10 weeks. *Methods* Eligible for this study were employed women aged between 18–65 years scheduled for a laparoscopic adnexal surgery and/or hysterectomy. Data were collected from patients, their supervisors and their gynaecologists, by means of electronic questionnaires during a 6 month follow-up period and an automatically generated, detailed weblog of the patient web portal (www.ikherstel.nl). Investigated process measures included: reach, dose delivered, dose received, and fidelity. In addition, attitudes towards the intervention were explored among all stakeholders. *Results* 215 patients enrolled in the study and accounted to a reach of 60.2 % (215/357). All intervention group patients used their account at least once and total time spent on the patient web portal was almost 2 h for each patient (median 118 min, IQR 64–173 min). Most patients visited the website several times (median 11 times, IQR 6–16). Perceived effectiveness among patients was high (74 %). In addition, gynaecologists (76 %) and employers (61 %) were satisfied with the web portal as well. Implementation of the second step of the intervention was suboptimal. Motivating patients to consent to additional guidance and developing an accurate return-to-work-prognosis were two important obstacles. *Conclusions* The results of this study indicate good feasibility for implementation on a broad scale of the e-health intervention for patients undergoing benign gynaecological surgery. To enhance the implementation of the second step of the perioperative care program, adaptations in the integrated care protocol are needed.

**Electronic supplementary material:**

The online version of this article (doi:10.1007/s10926-013-9475-4) contains supplementary material, which is available to authorized users.

## Introduction

In gynaecology—as in other surgical specialties—there is an increasing interest in accelerating recovery after conventional surgery as well as minimal invasive surgery. Although procedure costs may be higher in minimal invasive surgery than with more conventional approaches, there is a perception that minimal invasive surgery gains in cost-effectiveness through shorter length of hospital stay and quicker and better convalescence [1–3]. Reduction of inpatient stay can easily be measured and directly benefits a hospital financially. Convalescence, on the contrary, is not on top of the agenda of many health care policy makers. A reason might be the fact that convalescence is much more difficult to influence and monitor, especially now hospital stay is minimized and post-operative care is transferred to outpatient and primary care, and therefore, fragmented. In addition, there is a lack of recognised evidence-based convalescence recommendations for gynaecological procedures [4, 5], resulting in a situation in which structural convalescence recommendations regarding the resumption of (work)activities are mostly not provided at discharge, or when given, are based on tradition and anecdote [6–8].

The current poor organisation of peri-operative care in gynaecology may lead to delayed recovery, prolonged sick leave and higher risk of work disability [7, 9, 10] which is associated with a poorer quality of life [11, 12]. In addition, as women comprise 45 % of the workforce in the Netherlands [13], as well as in many other Western countries [14], the unnecessary absenteeism related to gynaecological procedures causes a considerable economic burden on society [11].

The ikherstel-study (“I recover-study”) is a randomized controlled trial (RCT) in which the effectiveness was evaluated of a multidisciplinary care program aimed at improving recovery and preventing delayed return to work following gynaecological surgery [15]. The intervention program, consisting of two steps, provides guidance to patients from the moment the surgery is planned until full resumption of all (work)-activities after the procedure. The intervention program was developed systematically, based on the intervention mapping protocol, involving all stakeholders in the development process [16, 17].

Besides developing an intervention systematically, it is of equal importance to evaluate the process of implementation systematically [18–20]. A good understanding of the extent to which the program was applied as intended, helps to interpret the outcome results in an effectiveness study. For example, in case positive effects of the program are not found, this could be attributable to either theory failure (the underlying theory is incorrect) or program failure (the program is potentially effective when implemented better) [21]. Moreover, a process evaluation helps to gain insight into the facilitators and barriers to future implementation which may expedite the challenging transition from research into daily practice.

This current paper describes the process evaluation of the intervention program of the ‘I recover-study’. The primary goal is to investigate the feasibility of the intervention by describing the process systematically. The second objective is to explore facilitators and barriers to future implementation.

## Methods

This process evaluation was carried out alongside a RCT studying the effectiveness of a multidisciplinary care program aimed at improving recovery and preventing delayed return to work following benign gynaecological surgery. The study design was approved by the Medical Ethics Committees of all participating hospitals and all participants signed informed consent. Details of the study design have been published elsewhere [15]. The effectiveness of the multidisciplinary care program was not evaluated in this feasibility study; these results will become available in the near future.

### Participants

All women aged between 18–65 years, employed for at least 8 h per week (salary-employed, self-employed or voluntary work) and scheduled for a surgery for benign gynaecological disease in one of the participating hospitals were eligible to participate. The types of surgeries that were included were: laparoscopic adnexal surgery (LAS) and/or total laparoscopic hysterectomy (TLH), vaginal hysterectomy (VH) or total abdominal hysterectomy (TAH). Excluded were patients with health problems or psychiatric disorders affecting daily life, as well as patients who were being sick-listed for more than 4 weeks prior to surgery or were involved in a lawsuit against their employer. Not being able to understand or complete the Dutch questionnaires, having no access to internet or internet-illiteracy were also exclusion criteria. This process evaluation was only performed for the participants randomised to the intervention group, because only they were exposed to the intervention care program.

### Recruitment

Waiting lists from participating hospitals were used to recruit prospective program participants. Patients were contacted by phone one week after they had received an invitation letter on behalf of their gynaecologist, together with an information package. Patients willing to participate and meeting the inclusion criteria were asked to return a signed informed consent. Patients were randomized to an intervention group (n = 110) or a control group (n = 105). As stated before, the current paper focuses only on the patients randomised to the intervention care program.

### Intervention

The intervention care program consists of a stepped care approach and contains two steps. The first step, an interactive e-health intervention, was provided to all participants in the intervention group. The second step, integrated care management, consisted of supplementary care coordinated by a clinical occupational physician and (if relevant) a workplace intervention by an occupational therapist (OT), and was only given to those participants whose sick leave exceeded 10 weeks.

The intervention care program was systematically developed applying the principles of intervention mapping [16]. Both theory and practise were combined and all stakeholders were involved in the process. The attitude, social influence and self-efficacy (ASE) model was used as a theoretical framework for determinants of behaviour regarding return to work (RTW) [22, 23]. Below, both steps of the program are summarized.

#### Step 1: E-Health Intervention

The e-health intervention http://www.ikherstel.nl was accessible to all patients, ideally 4 weeks prior to surgery. However, this period was shorter if the patient was enrolled closer to the surgery date. The patient web portal consisted of 47 unique pages and provided several tools aimed at empowering its users and improving communication between patients, employers and healthcare professionals during the peri-operative period. The most important tools are:

1. *Tool to compose reintegration plan* This tool enabled patients to generate detailed tailored instructions on the resumption of activities after the surgery. These recommendations were based on a multidisciplinary guideline developed by an expert panel of gynaecologists, general practitioners (GPs) and occupational physicians (OPs), using a structural consensus method prior to the RCT [24]. The tool was accessible before surgery, allowing planning of (work) activities and work reintegration. After surgery, the gynaecologist who had performed the surgery was asked to approve the reintegration plan electronically, allowing making adjustments to the standard advice in case of (surgical) complications.
2. *Video* A film was developed and available to watch on the patient web portal illustrating common pitfalls during the peri-operative and reintegration period.
3. *Tool to invite employer* Patients were stimulated to invite their employer to an (anonymous) section of the web portal, including the video. This tool aimed to improve communication between employee and employer and to stimulate to develop a reintegration plan (before surgery) and discuss potential RTW problems. For both the employee as the employer a list of recommendations was provided.
4. *Recovery monitor* Patients’ recovery was closely monitored by the patient web portal after surgery. At 2, 4, 7, 14, 28, 56 and 84 days after surgery, patients were encouraged to fill out the monitor, inventorying which activities they had resumed already and which they had not. If patients were not satisfied with their recovery or reintegration process, an alerting system advised them to contact a specific health professional, depending on the cause of dissatisfaction.
5. *Tools to increase knowledge and forum* Several tools were available to provide additional information, such an extended list with answers to frequently asked questions (FAQ), a glossary, and links to other useful patient web portals. In addition, there was a forum enabling patients to interact (privately or publicly) with other patients.

#### Step 2: Integrated Care Management

Integrated care management refers to a multidisciplinary approach to assist those patients who exceeded 10 weeks of sick leave. A clinical occupational physician was trained as RTW coordinator and fulfilled an intermediate role between the involved health professionals, including a trained occupational therapist (OT) and the patients’ own gynaecologist, general practitioner (GP), and occupational physician (OP). The integrated care protocol consisted of two steps:

1. *Consultation with clinical occupational physician* All patients exceeding 10 weeks of sick leave were offered a consultation with the clinical occupational physician in the 10th or 11th week after surgery. During the first contact the clinical occupational physician assessed the mental and physical condition of the patient and discussed the job profile and demands. Taking all factors into consideration, a treatment and reintegration plan with a RTW prognosis was made. If both the patient and her own OP agreed to the plan, the recommendations were executed by calling in the assistance of the OT (if relevant), the patients’ employer and/or appropriate health care provider(s).
2. *If necessary, participatory workplace intervention* When a patient was referred to the OT the workplace intervention procedure would start. The workplace intervention consists of three meetings: (1) OT with patient, (2) OT with supervisor and (3) OT, supervisor and patient together. The three meetings focus on identifying and prioritizing obstacles for RTW, finding solutions and achieving consensus between the patient and their supervisor with regard to work adjustments to facilitate RTW. The protocol was originally developed and proved effective for patients with chronic low back pain [25, 26] and is based on methods used in ‘participatory ergonomics’ [27]. The protocol was adapted to post-operative gynaecologic patients regarding time schedule and involved care providers.

### Data Collection

Data for this process evaluation were collected from the patients using online questionnaires at baseline and during the 6 month follow up (2, 6, 12 and 26 weeks after surgery). Besides data collection from the patients, we collected data from (1) the patients’ employers (online questionnaire at 8 weeks after surgery) (2) the patients’ gynaecologists (online questionnaire after the trial) and (3) the occupational physician involved in the study (evaluation interview after the trial). In addition, data were also obtained by means of an automatically generated weblog of the web portal.

### Process Measures

According to the recommendations of Linnan and Steckler [20] the following process items were assessed: (1) the context of the intervention, (2) reach, (3) dose delivered, (4) dose received, (5) fidelity and (6) participants’ attitudes towards the different steps of the intervention program. Table 1 gives an overview of these process measures.

**Table 1:** Process-measures, definitions and data-collection methods

Process measure	Step 1: E-health intervention	Step 2: Integrated care management
*Reach* Proportion of the target population that received the intervention	*Definition* Proportion of recruited potential participants that met all inclusion-criteria and decided to engage in the study	*Definition* Proportion of participants whose sick-leave exceeded 10 weeks that received consultation with OP
	*Data collection-method* Telephone-log Baseline-questionnaire	*Data collection-method* RTW-calendars Study database
*Dose delivered* Proportion of intended intervention that was actually delivered to target population	*Definition* Proportion of study population that received an account for the patient web portal	*Definition* Proportion of patients whose sick leave exceeded 10 weeks that received appointment with OP
	*Data collection-method* Weblog	*Data collection-method* Appointment system OP
*Dose received* Extent to which the participants used the intervention as recommended	*Definition* Proportion of patients with an account that used the web portal to compose a reintegration plan at least once	*Definition* Proportion of patients with an appointment that received a consultation and consented with the recommendations of the OP regarding follow-up
	*Data collection-method* Weblog	*Data collection-method* Patient records OP
*Fidelity* Extent to which the intervention was delivered as planned	*Definition* Proportion of patients who had their reintegration plan electronically approved by their gynaecologist	*Definition* Proportion of consultations that took place without violation of the study protocol (e.g. referral to participatory workplace intervention if sick leave exceeded 12 weeks)
	*Data collection-method* Weblog	*Data collection-method* RTW-calendars Patient records OP
*Participants’ attitudes* Satisfaction Perceived effectiveness Usage barriers Suggestions for improvement	*Target* Patients Gynaecologists Employers	*Target* Clinical occupation physician
	*Data collection-method* Online questionnaire	*Data collection-method* Face-to-face interview

*OP* clinical occupational physician, *RTW* return to work

#### Context of the Intervention

Context refers to the larger physical, social and political environment that can affect an intervention program. In this process evaluation we did not assess contextual influences, however, in order to consider future implementation of the intervention program, an understanding is needed of the Dutch social and political situation. Online Resource 1 provides a short overview on sickness benefit guidance in the Netherlands. In summary, employers are obliged to continue to pay wages of their employers during the first two years of sickness. During this two year period, both the employer as the sick listed employee share a mutual responsibility to increase the probability of return to work. If the employer fails to pursue an active absenteeism policy, he might be required to continue paying that employee’s salary for another year. However, if the employee hinders an early return to work, the payment of his sickness benefit may be suspended or reduced.

#### Reach

Reach concerns the degree to which an intended audience participated in the intervention.

Step 1: The e-health intervention was intended for all patients allocated to the intervention arm of the RCT. A detailed telephone log and the study database were used to determine what proportion of recruited potential participants did decide to engage in the study and who declined to participate. Reasons for exclusion were registered, as well as the number and reasons for drop-outs.

Step 2: Integrated care management was intended for only those patients whose sick leave exceeded 10 weeks. Return to work data were collected through the patient web portal as well as through monthly self-reported calendars of sickness absence. Retrospectively, the proportion could be determined of the patients actually receiving the second part of the intervention considering the total number of patients who should have received it.

#### Dose Delivered

Dose delivered refers to the proportion of the intended intervention that is actually delivered to the program participants and is determined by the actions of the intervention provider.

Step 1: Accounts for the patient web portal were provided by the research team. The number of generated accounts divided by the total number of participating patients was defined as dose delivered.

Step 2: According to the protocol, the clinical occupational physician should have offered a consultation to all patients exceeding 10 weeks of sick leave. Dose delivered was determined by the number of invitations divided by the total number of patients with extended sick leave.

#### Dose Received

Dose received is a measure of the extent to which participants actively engage with the intervention. For this paper dose received was defined as the proportion of patients that used the intervention as recommended by the health care providers, likewise the definition of adherence used by World Health Organization (WHO) [28].

Step 1: Activity on the patient web portal was continuously and automatically registered in a weblog. Because of user authentication (username and password) every participant had a unique ID, which made it possible to analyse website activity for each individual participant. Information stored in the weblog included visited page numbers, time stamps (start and end-time) and number of sessions. To prevent over-estimation of activity time, a timer was built in the system which stopped time registration when participants were not active (scrolling, click or mouse movement) for a period of 8 min. The minimum recommended use of the website was defined as usage of the tool to compose an integration plan at least once, as a tailored schedule with convalescence recommendations enables patients to plan their daily and work-activities after the surgery and to anticipate on facing problems as well. In addition, possible irrational beliefs about recovery could be rectified with this reliable source of information.

Step 2: For the integrated care management dose received was defined as the proportion of patients that received a consultation with the clinical occupational physician and who consented with the recommendations of the OP regarding follow-up, e.g. a referral for the workplace intervention.

#### Fidelity

Fidelity refers to the quality of the deliverance of an intervention and the extent to which the intervention was delivered as planned.

Step 1: Each gynaecologist who performed a surgical procedure on a participating patient received an electronic request to approve the reintegration plan that the patient had composed on the patient web portal. This essential step prevented that the standardized convalescence recommendations were given to patients with (surgical) complications. If thought relevant, the gynaecologist could adjust the recommendations, and the patient received a confirmation. If a patient experienced complications after discharge from the hospital, she could notify her gynaecologist through the web portal, and he or she was asked to review the patient’s reintegration plan again. Fidelity was defined as the proportion of patients whose reintegration plan was approved and/or adjusted by their gynaecologist.

Step 2: Fidelity for the integrated care management was determined by the number of consultations that took place without violation of the study protocol (e.g. accuracy of scheduled appointments, visits or telephone-consultations). Retrospectively, it was determined in how many cases a good assessment was made of the patient’s situation, and if the participatory workplace intervention was indicated correctly (sick leave >12 weeks).

#### Implementation Score

For each step of the care program an implementation score was calculated using the average of the four process measures.

#### Participants’ Attitude

Participants’ attitudes towards the e-health intervention were assessed among patients, gynaecologists and employers. Patients were requested to rate their satisfaction with the (different tools of the) patient web portal. In addition, perceived effectiveness was scored on a 5 point Likert Scale and patients were asked if they would recommend the e-health intervention to a friend (yes/no). Reasons for (non-)compliance were evaluated and patients could give suggestions for improvement.

Among employers satisfaction with the different items on the anonymous section of the web portal was assessed, as well as their satisfaction with the guidance the web portal offered their employee during the peri-operative period (both on 5 point Likert scale). Suggestions for improvement were evaluated.

Gynaecologists’ opinion on the feasibility of the e-health intervention was evaluated through named facilitators and barriers to future implementation and their answers to the question if they would offer the intervention to their patients if widely available (yes/no). Again, suggestions for improvement were registered.

The clinical occupational therapist involved in the study was asked about her experience with the integrated care management during an evaluation interview after the trial.

### Data Analysis

MATLAB version 7.1 (The MathWorks Inc., Natick, MA, USA) was used to transform the weblog into user and page statistics. SPSS version 20.0 (IBM Corporation, Amonk, NY, USA) and Excel 2003 (Microsoft, Washington, DC, USA) were used for descriptive and statistical analyses. Quantitative data were analysed by means of descriptive statistics such as frequencies, means, medians and interquartile ranges. To compare differences in groups, independent t-tests or Mann–Whitney *U* tests were used for continuous variables, depending on the distribution. All tests were performed two-sided. Statistical significance was defined as *p* < 0.05.

## Results

### Step 1 E-Health Intervention

#### Reach

**Fig. 1:**
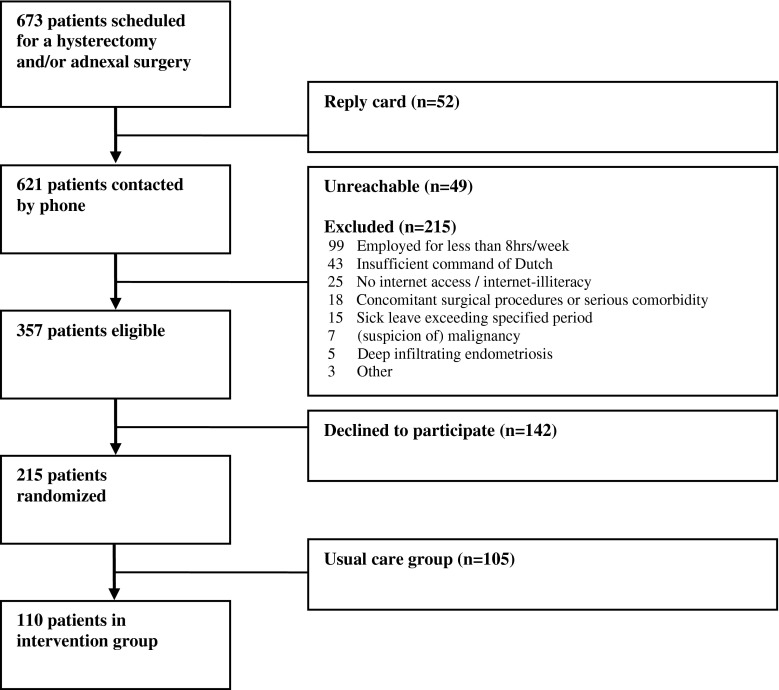
Study flow diagram

Between March 2010 and January 2011 a total of 673 patients were scheduled for a hysterectomy and/or laparoscopic adnexal surgery in one of the participating hospitals. Fifty-two patients (7.7 %) returned the reply card which was included in the information package, indicating they were not interested in participation. Of the 621 patients to be contacted by telephone, 49 patients were unreachable and 215 patients were excluded because they did not meet the inclusion criteria of the study. The main reason for exclusion was the lack of employment or working less than 8 h a week (99/215; 46 %). A total of 357 patients were eligible for the study, of which 142 patients declined to participate. The remaining 215 patients enrolled in the study and accounted to a reach of 60.2 % (215/357). Figure 1 shows a flow-diagram of the study participants.

Randomization was performed after informed consent and the baseline measurement. The present paper, only reports on the participants allocated to the intervention group (110 patients). Table 2 shows the baseline characteristics of these participants. These participants did not differ significantly from the patients who were allocated to usual care.

**Table 2:** Baseline characteristics–intervention group (n = 110)

Patient characteristics	
Age (years ± SD)	43.5 ± 7.8
Education level^a^	
Low	10 (9.1)
Intermediate	50 (45.5)
High	50 (45.5)
Surgery related characteristics	
Adnexal surgery (LAS)	51 (46.0)
Laparoscopic hysterectomy (TLH)	17 (15.5)
Vaginal hysterectomy (TVH)	25 (23.0)
Abdominal hysterectomy (TAH)	17 (15.5)
Health related characteristics	
Self-rated health status (mean ± SD)^b^	78.4 ± 15.7
Work related characteristics	
Type of work	
Salaried employed	89 (80.9)
Self-employed	19 (17.3)
Voluntary work	2 (1.8)
Work hours per week (mean ± SD)	30.3 ± 9.2

Numbers present frequencies and percentages unless otherwise specified

^a^Low = preschool, primary school; intermediate = lower and upper secondary; high = tertiary education, university or postgraduate

^b^EuroQol VAS-scale ranging from 0 (=worst imaginable health) to 100 (=best imaginable health)

The primary outcome full sustainable return to work was complete for all participants. The questionnaires assessing secondary outcome measures at 2, 6, 12, and 26 weeks were completed by 93.6 to 95.6 % of all participants.

#### Dose Delivered

All 110 patients were given access to the patient web portal www.ikherstel.nl before their surgery by the principal investigator or research-assistant (dose delivered: 100 %). The median number of days patients accessed the web portal prior to their surgery was 16 days (IQR 9–29 days). In 12.7 % of the cases, patients were given access only a week prior to the surgery. These cases can be explained because surgeries were planned on short notice or patients failed to complete the baseline questionnaire earlier.

#### Dose Received

Table 3 presents data about the usage of the patient web portal and the different tools. All patients used their account at least once, with the vast majority (98.8 %) doing this before surgery. Total time spent on the patient web portal by each patient was almost 2 h (median 118 min, IQR 64–173 min) (Table 3). Most patients visited the website several times with a median number of 11 sessions (IQR 6–16).

**Table 3:** Patient use of web portal

Overall	Total visit duration per patient (minutes)	118 (64–173)
	Number of sessions	10.5 (6–16)
	First login before surgery	108 (98.2 %)
	First login after surgery	2 (1.8 %)
	≤2 sessions	7 (6.4 %)
	>2 sessions	103 (93.6 %)
Website Tools	Reintegration plan	
	Composition before surgery	63 (57.3 %)
	Composition after surgery	32 (29.1 %)
	No composition	15 (13.6 %)
	Video	
	Number of unique visitors	77 (70.0 %)
	Total visit duration per patient (minutes)	8.9 (3.9–11.4)
	Interaction with employer	
	Number of invitations^a^	41 (46.1 %)
	Number of unique visitors to page displaying recommendations for employee	73 (66.4 %)
	Number of unique visitors to page displaying recommendations for employer	55 (50.0 %)
	Recovery monitor	
	Number of unique visitors	106 (96.4 %)
	Total visit duration per patient (minutes)	46.2 (28.5–69.8)
	Number of visits per patient	13 (10–16)
	Frequently Asked Questions	
	Number of unique visitors	58 (52.7 %)
	Total visit duration per patient (minutes)	9.3 (2.1–17.6)
	Forum	
	Number of unique visitors	61 (55.5 %)
	Total visit duration per patient (minutes)	2.2 (0.9–6.5)
	Number of visits per patient	6 (3–15)

Numbers present frequencies (%) or medians (IQR)

^a^Only relevant for patients with an employer (n = 89)

**Fig. 2:**
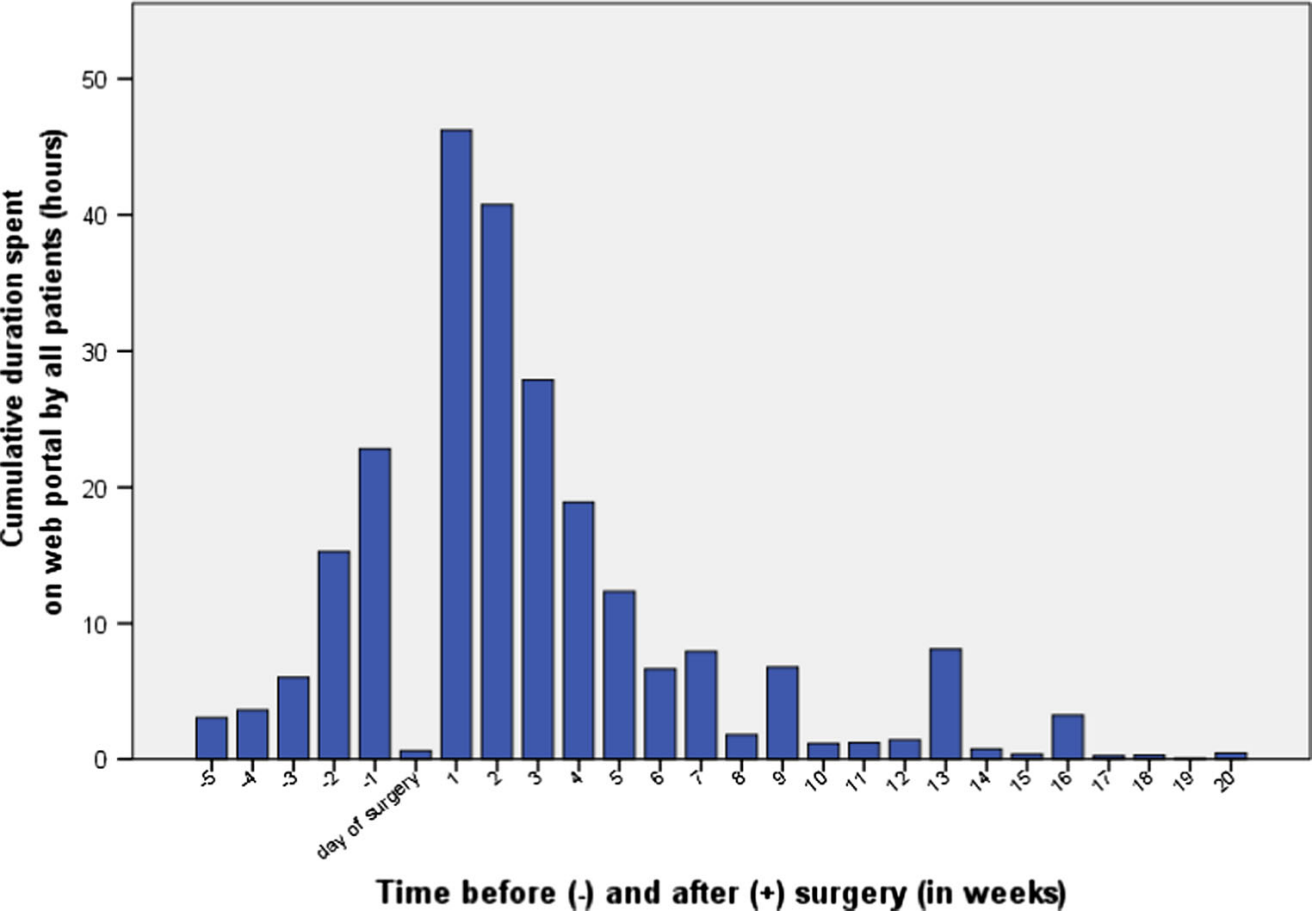
Use of patient web portal related to date of surgery

Activity on the patient web portal was highest in the week before surgery and the first 3 weeks after surgery (Fig. 2). An average session lasted 12 min and 15 pages were viewed per session. There was no significant statistical difference in usage of the patient web portal between patients undergoing different types of surgery.

Before surgery, 63 patients (57.2 %) used the tool to compose a reintegration plan. Taken the total follow up into account, the majority of patients used the tool (dose received: 95/110; 86.4 %).

#### Fidelity

Reintegration plans were electronically approved in 3 out of every 4 patients accounting to a fidelity score of 74.5 % of all cases (82/110). In 25 remaining cases (22.7 %), the principle investigator approved the schedules after having had contact with the surgeon. Reasons given by surgeons for not approving the schedule themselves were: lack of time, loss of electronic invitation or sudden change of surgeon. In 7 cases the surgeon adjusted the standard reintegration schedule because of complications during or after the surgery.

#### Implementation Score

Using the average of the four process-measures, the implementation score of the first step of the intervention was 80.3 % ((60.2 + 100 + 86.4 + 74.5 %)/4).

#### Participants’ Attitudes Towards the Intervention

*Patients* Satisfaction-scores with the different tools of the website are presented in Table 4. The vast majority of patients (75/102; 73.5 %) were (very) satisfied with the tool to compose a reintegration plan and found it (very) useful to plan normal activities (67.6 %) and work-activities (56.8 %). The majority of patients (87/105; 82, 9 %) followed most convalescence recommendations. Twelve patients explained they did not need a schedule because they rather resumed activities when their body felt ready for it. Another reason for non-compliance was finding the reintegration schedule too optimistic (23 times), while others stated the recommendations were too conservative (12 times).

**Table 4:** Satisfaction with different tools of patient web portal

	Degree of satisfaction 1 = totally dissatisfied 5 = very satisfied					
	1	2	3	4	5	Not applicable
Patients (n = 102)						
Graded activity schedule for general well-being^a^	2.0	5.9	18.6	32.4	41.2	–
Graded activity schedule for planning normal activities^a^	3.9	6.9	21.6	39.2	28.4	–
Graded activity schedule for planning work related activities^a^	5.9	11.8	25.5	33.3	23.5	–
Links to other websites	1.0	1.0	28.4	33.3	6.9	29.4
Forum	5.9	4.9	26.5	16.7	3.9	42.2
FAQ	1.0	1.0	25.5	40.2	9.8	22.5
Film	2.9	3.9	32.4	29.4	2.9	28.4
Employers (n = 26)						
Film	7.7	0.0	19.2	30.8	11.5	30.8
Recommendations for patients	0.0	0.0	23.1	42.3	7.7	26.9
Recommendations for employers	0.0	7.7	30.8	42.3	7.7	11.5

Numbers present percentages

*FAQ* frequently asked questions

^a^Obligatory choice of score 1 to 5

Perceived effectiveness of the e-health intervention was high. At 12 weeks, 73.5 % (75/102) of all participants felt usage of the web portal contributed positively to their recovery. People who did not perceive an additional effect explained they did not need the web portal (8 times), they felt pushed by the convalescence advice (5 times) or they felt the e-health intervention did not apply to their personal situation (4 times). Eighty-seven patients (87/102; 85.3 %) would recommend the web portal to a friend. Suggestions for improvement included an extra section with experiences of other women (3 times).

*Employers* Almost half of the salary-employed participants invited their employer to visit an anonymous section of the website (42/89; 47.2 %). Reasons given for not using this tool included: finding it unnecessary because of a fast recovery or good relationship with employer (16 times), not wanting to be a burden or anticipating the employer not to be interested (8 times) or not wanting to share private information with their employer (5 times). Satisfaction about guidance provided by their employer did not differ statistically between patients who did and patients who did not invite their employer.

Twenty-six employers (63.4 %) completed the digital questionnaire 8 weeks after the surgery of their employee. Satisfaction-scores with the different tools offered by the web portal are presented in Table 4. In total, 61.1 % of the employers (11/18) were (very) satisfied with the guidance the web portal offered to their employee. One employer suggested including extra information about reintegration-schedules.

*Gynaecologists* In total, 40 gynaecologists were involved in the study, with a median number of 2 patients each (range 1–9). Thirty-one gynaecologists (77.5 %) finished (part of) an electronic questionnaire at the end of the trial. Of the 28 gynaecologists answering the questions about usefulness of the intervention, seven gynaecologists found themselves unable to give an answer because of too little experience with the intervention. Of the remaining 21 gynaecologists, 76.2 % rated the e-health-intervention as (very) useful (16/21). The vast majority would offer it to their patients, would it be widely available (20/21; 95.2 %).

Possible future usage barriers for patients included: required access to internet (3 times) and the inflexibility of the e-health intervention in case of complications (2 times). Possible usage barriers for gynaecologists were an increased time-investment (7 times). However, only 2 gynaecologists (2/28; 7.1 %) were unsatisfied with their own actual time-investment in delivering the intervention.

### Step 2 Integrated Care Management

#### Reach

At 10 weeks after surgery 25 patients (25/110; 22.7 %) had not fully returned to work and represented the target audience for the second part of the intervention program, the integrated care management. In total, 12 consultations with the clinical occupational physician took place, accounting for a reach of 48 % (12/25).

As expected, patients with less invasive surgeries were more likely to have resumed their work-activities than those with more invasive surgeries. For the different types of surgeries the proportion of patients eligible for a consultation with the clinical occupational physician (OP) was as follows: TAH: 53 % (9 out of 17), VH: 28 % (7 out of 25), TLH: 29 % (5 out of 17), and LAS: 8 % (4 out of 51). In this group of delayed recovery, five patients (5/25, 20 %) suffered from a complication during or related to the surgery. Complications were defined as an enlargement of the wound with >8 centimetre or re-surgery within two weeks after initial surgery.

#### Dose Delivered

When patients had not resumed their work-activities 8 weeks after surgery, information about the integrated care management appeared on the patient web portal. Simultaneously, the clinical occupational therapist received the contact information of these patients and approached them by telephone to schedule an appointment in the 10th or 11th week after surgery.

In total, 17 appointments were scheduled, resulting in a dose delivered of 68 % (17/25). In two cases patients were not considered eligible for a consultation, due to medical reasons (severe complications related to the gynaecologic surgery) or personal reasons (recent death of partner). Six patients declined a consultation because they had already partly resumed their work activities and expected to fully return to work shortly. Four of them did resume completely within 12 weeks after surgery. Return to work of the last two patients took much longer than expected (16 weeks).

#### Dose Received

Of the 17 scheduled appointments, 12 consultations took place. Two patients cancelled because they had fully returned to work before the appointment and three patients cancelled because they did not feel the need for a consultation anymore. Given reason were: (1) the patient had partially resumed, (2) the patient had already consulted her own occupational physician, and (3) the patient did not wish to re-schedule the appointment when the clinical occupational therapist was forced to cancel the appointment.

Of the 12 consultations, 2 patients turned out to be sick-listed for other reasons than the gynaecologic surgery at time of the appointment (personal problems due to broken relationship and longer existing shoulder complaints). Two patients decided to decline further guidance from the OP during the first consultation. They did not disclose their reasons; however, they stayed sick-listed for 17 and 24 weeks respectively. Lastly, two patients declined a referral for the workplace intervention after discussing this treatment option with their supervisor and/or own occupational physician. One patient expected no additional benefit because she was satisfied with the guidance offered by her own occupational physician. The last patient experienced the consultation as unpleasant, because she felt pushed to return to work, while she felt she was not ready yet and therefore declined follow-up. Both patients stayed sick-listed during the complete follow up of 6 months.

In six cases follow up or referral to the occupational therapist was not indicated by the clinical occupational therapist because of a good RTW-prognosis. In these cases, the patients were already partially resuming their work-activities and did receive sufficient guidance from their own occupational physician and employer. Considering all consultations that were scheduled, the dose received calculated was 24 % (6/25) because in six consultations care was delivered according to the protocol.

#### Fidelity

The fidelity of the six remaining consultations was very poor (0 %). In all cases in which follow-up or a referral to the occupational therapist was not considered relevant, the good RTW prognosis was incorrect retrospectively. Average time to full RTW after the consultation with the clinical occupational physician was still more than 2 months (mean 66 days; range 40–78) with one participant not reaching full RTW at all. Further guidance of the clinical occupational therapist in these cases would probably have been beneficial. Moreover, only 3 patients visited the clinical occupational physician, the other nine consultations took place by telephone. Telephone consults were offered because patients were not willing to pay an actual visit because of the investment of time and money. In addition, only 3 cases were scheduled in the 10th or 11th week after surgery as indicated by the protocol, with 4 appointments scheduled too early (week 9) and 5 appointments too late (week 13–15).

#### Implementation Score

The implementation score of the second step of the intervention program was calculated to be 35 % ((48 + 68 + 24 + 0 %)/4).

#### Experiences of Clinical Occupational Physician

At the end of the trial the clinical occupational physician involved in the study was interviewed to evaluate the integrated care management. The most important topics discussed included the high number of patients that declined additional care and the difficulty to estimate RTW-prognosis. Moreover, possible solutions to these barriers were reviewed.

The clinical occupational physician explained she experienced most difficulties persuading participants to schedule an appointment with her. Because she met patients relatively late after the surgery, most patients were already partly resuming their work-activities and had already made a reintegration-plan often with help of their supervisors or own OPs. It was then very difficult to explain the additional value of a consultation, and in case of an appointment, make alterations in the plans already made. Secondly, most consultations took place by telephone, because patients were not willing to make a visit, making it very hard to develop an accurate RTW-prognosis.

In order to enhance the impact of a consultation, the clinical occupational physician advised to incorporate the consultation in standard care, e.g. women who are planned for a surgery should automatically receive an invitation for the clinical occupational physician. In addition, the moment of contact should be at a much earlier stage, even maybe before surgery, to be able to support the development of a solid RTW-plan and to influence irrelevant cognitions about their recovery. In the current format, the occupational physician was doubtful about the effectiveness of this part of the intervention.

## Discussion

### Main Findings

The aim of this paper was to evaluate the implementation process and experiences with an innovative care program for women undergoing benign gynaecological surgery. As the care program consisted of two different steps—an e-health intervention and integrated care management—both steps were evaluated separately, using the criteria outlined by Linnan and Steckler [20]. Overall, the e-health intervention was implemented fairly well with an implementation score of 80 %. Patients, gynaecologists and employers were all highly satisfied with the web portal www.ikherstel.nl. The implementation of the integrated care management protocol was less successful with a final implementation score of 35 %. Convincing patients about the additional value of a consultation with the occupational physician and developing an accurate RTW-prognosis were the two most important obstacles for the second step of the intervention program.

### Interpretation of the Findings

#### Step 1 E-Health Intervention

The use of e-health technologies is considered to be an important key to improving efficiency and quality of health care [29, 30]. Possible benefits include enhancing (self-) monitoring activities, increasing delivery of care based on guidelines, and decreasing utilization of health services. However, there remains a gap between the postulated and empirically demonstrated benefits [29]. The current process evaluation is an essential step towards improving implementation of evidence-based e-health interventions. To the best of our knowledge, our patient web portal is the first evaluated e-health intervention in both fields of postoperative care and gynaecology.

The reach of the e-health intervention was moderately high (60 %). In total, only 25 women were excluded because of having no access to the internet or internet-illiteracy (25/376; 3.7 %). In the Netherlands, the general internet-access rate is 96 % [31]. Compared to national numbers under working females, highly educated women were overrepresented in our study: 50 versus 35 % [32]. Partly, this might be explained by regional differences and the location of some hospitals in and near the capital of the Netherlands. However, selection bias might have played a role as well, when highly educated women might be more interested in the e-health intervention (and fast recovery) and decided to participate more often.

Compliance towards web-based interventions varies among different studies and target populations [33]. For depression and anxiety disorders adherence rates to online treatments are generally found between 50 and 70 % [34]. In our study we were able to objectively measure usage of the e-health intervention and 86 % of all participants used the web portal as intended. This is relatively high, but in concordance with the high satisfaction scores and an overall high perceived effectiveness of the e-health intervention.

#### Step 2 Integrated Care Management

Unfortunately, the second part of the intervention did not unfold and reasons might be found in the characteristics of the target population. Participatory workplace programs have been shown to be effective in patients sick-listed due to musculoskeletal disorders and distress [25, 35–37]. Generally, targeted patients were characterized by a history of chronic disease and complaints, whereas the target population in the current study consisted of patients working at the time of recruitment and facing only a temporary period of sick leave during the recovery of their surgery. This temporary nature of the sick leave is probably the most important barrier to full implementation, demonstrated by a number of issues. Firstly, more than half of the patients (13 out of 25) declined additional care at some time during the integrated care management, indicating a general lack of perceived value of additional guidance. This could be related to Dutch legislation which ensures salary income at least during the first 24 months of sick leave (see Online Resource 1). In absence of financial consequences, people might not be urged to return to work as soon as possible, and therefore less interested in initiatives to facilitate return to work. Moreover, a commonly given reason for rejecting a consultation was that the patient had already partly resumed and expected full return to work shortly. However, perception of the own situation turned out to be problematic as it took these patients still 3.5 months to resume all work activities after starting partly. Finally, developing an accurate RTW prognosis was challenging for the occupational physician as well (poor score on fidelity). Up to date, not much is known about prognostic factors for RTW in this specific population.

### Strengths and Limitations of this Study

A strength of this study is that data collection was performed systematically using an established theoretical framework to assess the process outcomes. Moreover, multiple sources were employed such as online questionnaires and the weblog generated from the patient web portal. The latter allowed a detailed and objective evaluation of patient compliance to the e-health intervention. Finally, all stakeholders of the intervention program (patients, employers, gynaecologists and the clinical occupational physician) were included in this process evaluation.

This study also has limitations. For example, we failed to measure contextual factors that might have influenced implementation. Moreover, we should be aware that a research setting can be advantageous towards an intervention, due to highly involved health professionals, motivated patients (selection bias) and interference of the research team. In the current study this can be illustrated by the artificial score of 100 % for dose delivered. Earlier research showed that adherence rates to open access websites can be much lower compared to a research environment (up to 50 % less) [33], so this needs to receive special attention when implementing the intervention program into daily practice. Some procedures that were carried out by the research team should be automated, such as generating accounts. Other procedures will have to be transferred to the health care providers. However, we presume the intervention to receive enough support, as 9 out of 10 gynaecologists indicated they would offer the intervention to their patients would it be widely available.

### Practical and Research Implications

A considerable large number of patients reported that the reintegration plan they had composed on the web portal was too optimistic for their own situation (23/110; 21 %). Some participants said this increased insecurities and anxiety, as they fell behind the schedule, which is a negative outcome of the intervention. Before broader implementation, it is essential to take measures to prevent this, as it will influence compliance negatively. The solution should not necessarily mean to loosen the convalescence recommendations, but could also be providing more information and targeting coping mechanisms.

Moreover, this process evaluation showed important directions to improve the second step of the intervention program and these lessons should be taken into account when implementing the intervention program on a wider scale. First of all, the importance of a prosperous recovery in means of improving quality of life and preventing long term sickness should be emphasized to patients. The patient web portal provides an excellent platform for this. In addition, possibilities to incorporate a consultation with a clinical occupational physician in standard care should be explored with all involved stakeholders. Possibly, patient’s own occupational physicians can perform this part of the intervention themselves in the future, as this would also increase support in the direct environment of the patient. Contact with the patient in an early stage seems to be crucial to influence patients’ attitudes and (irrational) beliefs about their recovery.

## Conclusions

This current paper describes the process evaluation of a new intervention program to provide additional guidance during the perioperative period to gynaecological patients. The results of this study indicate good feasibility for implementation on a broad scale of the e-health intervention. Compliance, perceived effectiveness and satisfaction were high among patients. In addition, other stakeholders such as gynaecologists and employers, assessed the intervention as potentially very useful. To enhance the implementation of the second step of the perioperative care program, adaptations in the integrated care protocol are needed.

## Electronic supplementary material

Below is the link to the electronic supplementary material.

Supplementary material 1 (DOC 40 kb)
