# Supplementary material for: Process Evaluation of a Multidisciplinary Care Program for Patients Undergoing Gynaecological Surgery
Source: J Occup Rehabil. 2013 Sep 22;24(3):425–38. doi: 10.1007/s10926-013-9475-4 (PMC4118044; doi:10.1007/s10926-013-9475-4)
Supplement: Supplementary file 1 — Supplementary material 1 (DOC 40 kb) [file 10926_2013_9475_MOESM1_ESM.doc]

**Online Resource 1**

| Article Title | Process evaluation of a multidisciplinary care program for patients undergoing gynaecological surgery |
| --- | --- |
| Journal | Journal of Occupational Rehabilitation |
| Authors: | E.V.A. Bouwsma, A. Vonk Noordegraaf, Z. Szlavik, H.A.M. Brölmann, M.H. Emanuel, J.P. Lips, W. van Mechelen, A. Mozes, M.D., A. L. Thurkow, J.A.F. Huirne, J.R. Anema |
| Corresponding author | E.V.A. Bouwsma  Department of Public and Occupational Health, VU University Medical Center, EMGO Institute for Health and Care Research, Amsterdam, The Netherlands  Department of Obstetrics and Gynaecology, VU University Medical Center, Amsterdam, The Netherlands  [Ev.bouwsma@vumc.nl](mailto:Ev.bouwsma@vumc.nl) |

**Sickness benefit guidance in the Netherlands**

In the Netherlands, employers are obliged to continue to pay –at least 70% of– the salaries of sick employees during the first two years of sickness. According to the Gatekeeper Improvement Act (April 2002) during this two year period, both the employer as the sick listed employee share a mutual responsibility to increase the probability of return to work. Both the employer as the employee may be sanctioned in case of noncompliance.

When an employee is sick listed for six weeks a reintegration report should be opened, which starts with a consultation with a company doctor (occupational physician - OP) of the official Health and Safety Executive Organisation (‘arbodienst’). The OP assesses the situation and makes a problem analysis, containing all the information relevant to the recovery, return to work and reintegration of the employee. Within two weeks, the employer and employee will then draw up a plan of action based on the concrete recommendations provided by the OP, which will be evaluated regularly, at least once every six weeks. Further consultations with the OP find place regularly as well.

The UWV (Institute for Employee Benefit Schemes) is the body commissioned by the Dutch Ministry of Social Affairs and Employment (SZW) to implement employee insurance schemes and acts as gatekeeper. When an employer did not reintegrated into the employment process within the two year period the UWV assesses if both parties have done everything possible to improve the chances of returning to work, by studying the total reintegration file. When both parties did make enough efforts, the employee can apply for a sickness benefit under the Work and Income according to Labour Capacity Act (WIA). However, if the employer failed to pursue an active absenteeism policy, sanctions may follow such as continuation of payment of the employee’s salary. On the other side, if the employee hindered an early return to work, the payment of his sickness benefit may be suspended or reduced.

Workers without an employer are granted a benefit for two years under the Sickness Benefit Act, also provided by UWV. In these cases, UWV is responsible for sickness absence counselling and reintegration as well.
